# Comparison of Patient-Controlled Analgesia With Tramadol or Morphine After Video-Assisted Thoracoscopic Surgery in Geriatric Patients

**DOI:** 10.7759/cureus.20781

**Published:** 2021-12-28

**Authors:** Gulay Ulger, Ramazan Baldemir

**Affiliations:** 1 Anesthesiology and Reanimation, University of Health Sciences, Ankara Atatürk Chest Diseases and Thoracic Surgery Training and Research Hospital, Ankara, TUR

**Keywords:** patient-controlled analgesia, video-assisted thoracoscopic surgery, pain, morphine, geriatrics, tramadol

## Abstract

Background

Although video-assisted thoracoscopic surgery (VATS) is a less invasive technique compared to thoracotomy, patients often experience postoperative pain. Hence, intravenous patient-controlled analgesia (PCA) is frequently used. The geriatric age group constitutes a significant portion of patients undergoing thoracic surgery. However, pain management can often be difficult in elderly patients. In this study, we aimed to examine the pain management techniques applied in geriatric patients who underwent VATS and to compare the efficacy and side effects of PCA with morphine and tramadol.

Methodology

The following patients were included in this study: aged 65 years and older, those who underwent elective VATS under general anesthesia, and those who underwent thoracic paravertebral block in the operating room for postoperative pain. We recorded diagnoses, demographic data, American Society of Anesthesiologists status, complications developed during the intraoperative or postoperative 24 hours, postoperative rest and cough Visual Analog Scale (VAS), and need for additional analgesics. The patients were divided into the following two groups: those treated with tramadol PCA (tramadol group) and those treated with morphine PCA (morphine group).

Results

A total of 65 patients were included in this study. Overall, 22 patients were administered tramadol PCA while 43 were administered morphine PCA. There was no statistically significant difference between the groups concerning complications. The 24-hour VAS resting score was statistically significantly lower in patients administered morphine than those administered tramadol (p < 0.05). There was no statistically significant difference between the groups concerning zero-minute, thirty-minute, one-hour, two-hour, six-hour, and twelve-hour VAS resting and cough scores at all times (p > 0.05).

Conclusions

There was no significant difference in the tramadol and morphine groups concerning analgesic efficacy, patient satisfaction, and side effects among geriatric patients who underwent VATS and were administered intravenous PCA. In our view, both tramadol and morphine can be used safely in geriatric patients requiring intravenous PCA. Moreover, because the 24-hour analgesic efficacy was observed to be better in the morphine group in our study, morphine can be preferred in geriatric patients.

## Introduction

Although thoracotomy is a frequently employed procedure in thoracic surgery, video-assisted thoracoscopic surgery (VATS) techniques are becoming widespread due to recent technological developments. VATS is applied as a reliable procedure in many thoracic surgeries [1-5]. Although it is a less invasive technique than thoracotomy, patients often experience postoperative pain [6]. If adequate postoperative analgesia is not administered, undesirable consequences such as atelectasis, decreased patient comfort, increased postoperative morbidity, prolonged hospital stay, and chronic pain may be encountered [6,7]. Peripheral nerve blocks are often preferred to prevent postoperative pain due to their low side effects [8]. However, intravenous analgesia is often combined with peripheral blocks to provide more effective analgesia [9]. Hence, intravenous patient-controlled analgesia (PCA) is frequently used. Morphine and tramadol are often preferred in patients undergoing intravenous PCA [10,11].

The geriatric age group constitutes a significant portion of patients undergoing thoracic surgeries. However, pain management can be often difficult in elderly patients [12]. Due to morbidity and pharmacokinetic and pharmacodynamic changes that occur with aging in elderly patients, drug use may increase side effects [7,12]. Studies have compared the analgesic efficacy and side effects of morphine and tramadol in patients undergoing intravenous PCA. However, there is no consensus on this issue [10]. Therefore, further studies comparing PCA with morphine or tramadol in geriatric patients undergoing VATS need to be conducted.

In this study, we aimed to examine the pain management techniques applied in geriatric patients who underwent VATS and to compare the efficacy and side effects of PCA with morphine and tramadol.

## Materials and methods

Following the approval of the Ethics Committee (Scientific Studies Commission, Date: 18.11.2021, Number: 13), this retrospective study was conducted in a tertiary chest diseases center between January 2020 and November 2021. Data were obtained by examining pain forms and operating room records of the anesthesia clinic. The following patients were included in this study: aged 65 years and older, those who underwent elective VATS under general anesthesia, those who underwent thoracic paravertebral block (TPVB) in the operating room for postoperative pain, and those who underwent postoperative intravenous PCA. The following patients were excluded from this study: those who underwent emergency surgery, those who underwent thoracotomy, those who underwent a peripheral block other than TPVB, those who did not undergo intravenous PCA, those who had a history of chronic pain, those who used continuous analgesic medications, and patients with missing data.

We recorded the diagnoses, gender, age, body mass index (BMI), American Society of Anesthesiologist (ASA) status, complications developed during the intraoperative or postoperative 24 hours (nausea, vomiting, hypotension, bradycardia, constipation, and respiratory depression), postoperative rest and cough Visual Analog Scale (VAS), the need for additional analgesics, and patient satisfaction with pain management.

**Figure 1:**
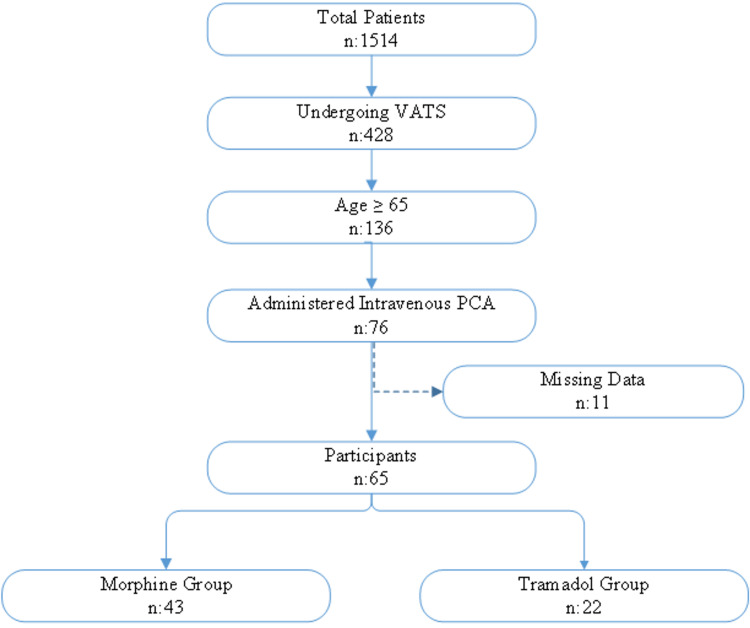
Flowchart of the patients included in this study. PCA: patient-controlled analgesia; VATS: video-assisted thoracoscopic surgery

The VAS scores during rest (VAS rest) and coughing (VAS cough) were measured at the postoperative zero hour (pre-PCA), thirty minutes, one hour, two hours, six hours, twelve hours, and twenty-four hours. VAS scores ranged from 0 (no pain) to 10 (maximum pain). The global pain rating was evaluated as good (VAS ≤ 3), moderate (4 ≤ VAS ≤ 6), and poor (VAS ≥ 7). The patients were divided into the following two groups: those treated with tramadol PCA (tramadol group) and those treated with morphine PCA (morphine group) (Figure 1).

The standard general anesthesia, peripheral nerve block, and analgesia protocol applied in the clinic

General Anesthesia

Written informed consent was obtained from the patients 24 hours before the operation. Patients were monitored in the operating room in accordance with the ASA standards, and intravenous 0.03 mg/kg midazolam was administered for premedication. After preoxygenation, anesthesia was induced intravenously using 1.5-2.5 mg/kg propofol, 1.5 µg/kg fentanyl, and 0.1 mg/kg vecuronium. Patients were intubated with a double-lumen endobronchial tube. Anesthesia was maintained by administering 2-3% sevoflurane in an oxygen and air mixture.

The Block Procedure

The block procedure was performed prior to the skin incision and following the induction of anesthesia. The TPVB was performed by injecting 20 mL of 0.25% bupivacaine into the paravertebral area with the patients in the lateral decubitus position.

Analgesia Protocol

Postoperatively, patients were given intravenous morphine or tramadol infusion using a PCA pump for 24 hours. Paracetamol (1 g) every eight hours and dexketoprofen (50 mg) twice daily were administered intravenously. As a rescue analgesic agent, 50 mg tramadol was given to patients intravenously when the VAS score at rest was greater than or equal to 4.

Patient-Controlled Anesthesia for the Morphine Group

Dose delivery of the PCA pump was limited to administering a bolus dose of 1 mg of morphine and delivering a maximum total dose of 12 mg of morphine over four hours at 15-minute lock-in intervals.

Patient-Controlled Anesthesia for the Tramadol Group

A concentration of 4 mg/mL tramadol was achieved. It was then adjusted to 10 mg/hour basal infusion, 10 mg bolus, 20-minute lock-in time, and a four-hour limit of 100 mg.

Statistical analyses

Data analyses were performed using SPSS for Windows, version 22.0 (IBM Corp., Armonk, NY, USA). Whether the distribution of continuous variables was normal was determined by the Kolmogorov-Smirnov test. Levene test was used for the evaluation of the homogeneity of variances. Unless specified otherwise, continuous data were described as mean ± standard deviation (SD) and median (1. quartile - 3. quartile) for non-normal distributions and skewed distributions, respectively. Categorical data were described as the number of cases (percentage). Statistical differences in non-normally distributed variables between two independent groups were compared using the Mann-Whitney U test. Categorical variables were compared using Pearson’s chi-square test or Fisher’s exact test. P-values of <0.05 were considered statistically significant.

## Results

VATS was employed in 428 of 1,514 patients who underwent thoracic surgery between January 2020 and November 2021. Overall, 136 (31.8%) of these patients were aged 65 or older. Among the elderly patients who underwent VATS, PCA was administered in 76 patients. In total, 11 patients were excluded from the study due to missing data. A total of 65 patients were finally included in this study. PCA with tramadol was administered in 22 patients and with morphine in 43 patients.

The demographic data of the patients according to the groups, the diagnoses and the type of surgery performed, the ASA physical status scores, and the number of patients who underwent rescue analgesia are presented in Table 1.

**Table 1:** Demographic data, diagnoses and type of surgery performed, ASA status, and the number of patients who underwent rescue analgesia. Continuous data are described as mean ± SD for non-normal distributions. Categorical data are described as the number of cases (%). ^β^: Mann-Whitney U test; ^Φ^: Pearson’s chi-square test or Fisher’s exact test; level of significance: p < 0.05. ASA: American Society of Anesthesiologists; BMI: body mass index; SD: standard deviation

		Tramadol group (n = 22)	Morphine group (n = 43)	P-value
		n (%)	n (%)	
Age (year)		70.73 ± 5.59	70.40 ± 4.01	0.797^β^
Gender	Female	5 (22.7%)	8 (18.6%)	0.749^Φ^
	Male	17 (77.3%)	35 (81.4%)	
BMI (kg/m^2^)		27.58 ± 4.20	26.73 ± 4.44	0.360^β^
Diagnoses/Surgery	Mass (lobectomy/wedge resection)	15 (68.2%)	22 (51.2%)	0.116^Φ^
	Bullous lung (wedge resection)	3 (13.6%)	3 (7.0%)	
	Pleural effusion (pleural biopsy)	4 (18.2%)	18 (41.9%)	
ASA	ASA II	4 (18.2%)	6 (14.0%)	0.814^Φ^
	ASA III	17 (77.3%)	33 (76.7%)	
	ASA IV	1 (4.5%)	4 (9.3%)	
Number of rescue analgesia patients		6 (27.3%)	14 (32.6%)	0.662^Φ^

Nausea/vomiting, respiratory arrest, and constipation were not seen in either group. While hypotension was observed in one patient in the tramadol group, bradycardia was observed in one patient in the morphine group. There was no statistically significant difference between the groups concerning complications (Table 2).

**Table 2:** Complications noted in patients. Categorical data are described as the number of cases (%). Φ: Pearson’s chi-square test or Fisher’s exact test; level of significance: p < 0.05.

Complication	Tramadol group (n = 22)	Morphine group (n = 43)	P-value
	n (%)	n (%)	
Hypotension	1 (4.5%)	0	0.338^Φ^
Bradycardia	0	1 (2.3%)	0.999^Φ^

The global pain assessment findings of the patients are presented in Table 3.

**Table 3:** Global pain assessment results of patients. Categorical data are described as the number of cases (%). ^Φ^: Pearson’s chi-square test or Fisher’s exact test; level of significance: p < 0.05.

Global pain assessment	Tramadol group (n = 22)	Morphine group (n = 43)	P-value
	n (%)	n (%)	
Good	15 (68.2%)	28 (65.1%)	0.999^Φ^
Moderate	5 (22.7%)	11 (25.6%)	
Poor	2 (9.1%)	4 (9.3%)	

**Figure 2:**
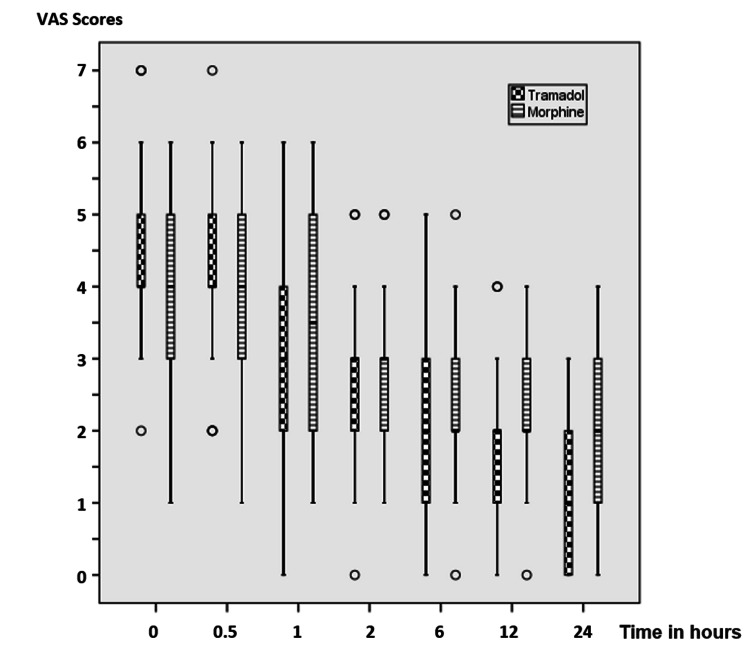
VAS rest scores of patients. Data are expressed as median (horizontal bar), interquartile range (box), maximum and minimum values (whiskers), and outliers (circles) for VAS scores. VAS: Visual Analog Scale

**Figure 3:**
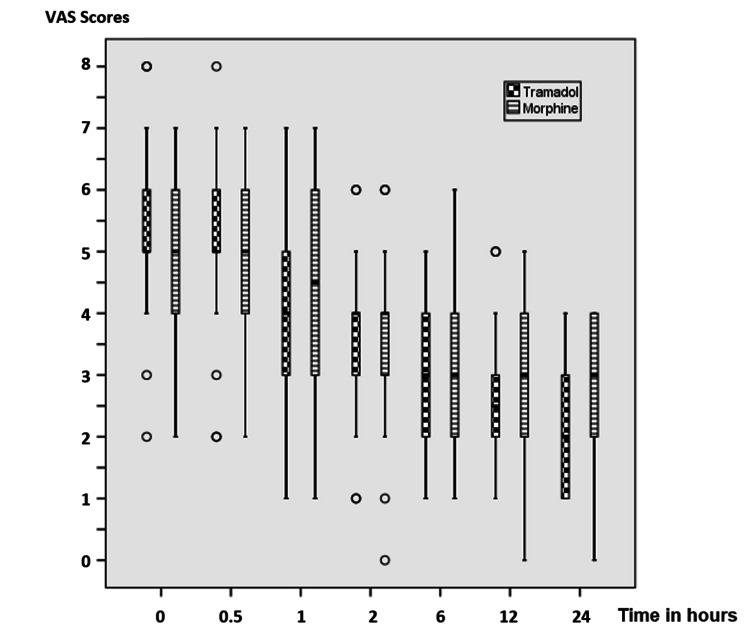
VAS cough scores of patients. Data are expressed as median (horizontal bar), interquartile range (box), maximum and minimum values (whiskers), and outliers (circles) for VAS scores. VAS: Visual Analog Scale

The 24-hour VAS rest score was statistically significantly lower in patients administered morphine than those administered tramadol (p < 0.05). There was no statistically significant difference between the groups concerning zero-hour, thirty-minute, one-hour, two-hour, six-hour, and twelve-hour VAS rest and cough scores at all times (p > 0.05) (Table 4, Figures 2, 3).

**Table 4:** VAS rest and cough scores of patients in the tramadol and morphine groups. Continuous data are described as mean ± SD for non-normal distributions. Mann-Whitney U test; level of significance: p < 0.05. SD: standard deviation; VAS: Visual Analog Scale

	Tramadol group (n = 22)	Morphine group (n = 43)	P-value
	Mean ± SD	Mean ± SD	
VAS rest			
0 minute	3.86 ± 1.52	3.93 ± 1.64	0.966
30 minutes	3.68 ± 1.62	3.70 ± 1.70	0.870
1 hour	3.36 ± 1.53	3.00 ± 1.69	0.413
2 hours	2.73 ± 1.20	2.51 ± 1.45	0.648
6 hours	2.32 ± 1.25	2.02 ± 1.32	0.512
12 hours	2.14 ± 0.99	1.63 ± 1.23	0.069
24 hours	1.95 ± 1.00	1.12 ± 1.14	0.005
VAS cough			
0 minute	4.86 ± 1.52	4.88 ± 1.69	0.977
30 minutes	4.68 ± 1.62	4.65 ± 1.76	0.814
1 hour	4.27±1.64	3.98 ± 1.73	0.512
2 hours	3.50 ± 1.50	3.51 ± 1.50	0.972
6 hours	3.23 ± 1.31	2.95 ± 1.21	0.529
12 hours	2.82 ± 1.30	2.58 ± 1.18	0.400
24 hours	2.59 ± 1.26	2.21 ± 1.04	0.144

## Discussion

In this study, 31.8% of the patients who underwent VATS in the clinic were aged 65 years and older. In the clinic setting, intravenous PCA is mostly applied using morphine in patients over 65 years of age. There was no difference between morphine and tramadol PCA concerning nausea/vomiting, hypotension, bradycardia and respiratory depression, and global pain assessment. The 24-hour VAS rest score was lower in patients treated with morphine than patients who were administered tramadol.

Intravenous morphine is frequently used with PCA, especially in the treatment of postoperative pain [13]. However, there are some limitations in the use of morphine due to its possible side effects such as constipation and respiratory depression [14]. In contrast, tramadol is a centrally acting opioid-like drug with fewer side effects compared to morphine [10,15].

Clinicians are often reluctant to use opioids because of their potential side effects [16,17]. Additionally, in elderly patients, increased sensitivity to opioids due to pharmacodynamic and pharmacokinetic changes should be considered. Similarly, attention should be paid to the increase in the incidence of side effects that may be caused by the use of multiple drugs due to morbidity in elderly patients [12].

Inadequate postoperative pain management can lead to increased morbidity and mortality [18]. Additionally, inadequate pain management may result in decreased patient satisfaction, delayed mobilization, increased complications, prolonged hospital stay, and increased costs [19,20]. After thoracic surgery, early mobilization of patients and clearing of secretions with coughing in the lungs are crucial. Early mobilization and adequate coughing are possible with appropriate and adequate pain management. Thus, adequate pain control after thoracic surgery is essential to reduce postoperative pulmonary complications [21].

Studies have shown that intravenous PCA applications increase patient satisfaction in the postoperative period and are successful in the treatment of pain [22,23]. There are different results in the literature regarding the effectiveness of morphine and tramadol in intravenous PCA applications. Some studies have reported that the use of morphine for intravenous PCA is more effective than tramadol for the treatment of pain, and the need for additional analgesics in the tramadol group is higher than in the morphine group [10,24]. Another study reported that intravenous PCA administered with morphine and tramadol was similar in terms of postoperative pain scores and additional analgesic requirement [25]. However, these studies are not specific to the geriatric age group.

Treatment of pain is challenging in geriatric patients due to the risk of side effects such as lack of expression of pain, respiratory depression, and hypotension [7,12]. In our study, for the geriatric age groups, we found that the patients’ 24-hour VAS rest scores were lower in the morphine group than in the tramadol group. Moreover, there was no difference in terms of VAS cough and VAS rest scores after 24 hours. In addition, additional analgesic use was similar in both groups in our study.

Adjusting bolus doses in PCA applications is crucial for adequate pain management [10]. In one study, a 2.5 mg/kg intravenous tramadol loading dose followed by a 10 mg bolus dose and a 10-minute lock time was set for tramadol. In the same study, a loading dose of 0.1 mg/kg intravenous morphine was administered for morphine, followed by a 1 mg bolus dose and a 10-minute lock-in time. The analgesic efficacy was similar in both PCA groups [26]. In another study, a bolus dose of 1 mg for morphine and 30 mg for tramadol, a lock time of 10 minutes in both groups, and analgesic efficacy were found to be similar in both groups [27]. In our study, a basal infusion rate of 10 mg/hour was determined for geriatric patients for tramadol. In addition, a 10 mg bolus dose and 20-minute lock time were determined for tramadol. For morphine, the bolus dose was 1 mg and the lock time was 15 minutes. Except for the 24-hour VAS rest score, there was no difference between the groups in terms of analgesic effectiveness with the infusion and bolus protocols in our study. According to the 24-hour VAS rest score, the 24-hour analgesic activity was more successful in the morphine group.

Although studies have reported that tramadol causes less respiratory depression than morphine, PCA administered with morphine and tramadol, in general, has a similar side-effect profile [10,24,26-28]. In our study, hypotension was observed in only one patient in the tramadol group and bradycardia was observed in only one patient in the morphine group. No nausea/vomiting or respiratory depression was observed in either group. Therefore, the tramadol and morphine groups were similar in terms of the risk of side effects.

There are some limitations to our study. Because this was a retrospective study, we could not evaluate the post-24-hour pain scores of the patients from the records. Therefore, only the first 24-hour VAS data of the patients were included in the study. In addition, because only geriatric patients who underwent VATS were included and patients with missing data were excluded, the number of patients included in the study was limited. Another limitation is that our study was conducted at a single center.

## Conclusions

There was no significant difference in the tramadol and morphine groups concerning analgesic efficacy, patient satisfaction, and side effects for intravenous PCA administered patients in the geriatric age group who underwent VATS. In our view, tramadol and morphine can be used safely in geriatric patients scheduled to undergo intravenous PCA, and morphine can be preferred in geriatric patients because the 24-hour analgesic efficacy was better in the morphine group in our study.
